# P039: A new method to assess compliance with measures to prevent nosocomial influenza transmission

**DOI:** 10.1186/2047-2994-2-S1-P39

**Published:** 2013-06-20

**Authors:** A Iten, C Bonfillon, T Bouvard, C-A Siegrist, D Pittet

**Affiliations:** 1 Infection Control Program, University of Geneva Hospitals, Geneva, Switzerland; 2 Department of Health Employees, University of Geneva Hospitals, Geneva, Switzerland; 3 Department of Child and Adolescent Medicine, University of Geneva Hospitals, Geneva, Switzerland

## Introduction

Seasonal influenza (SI) can present a serious threat to some patients, particularly those with underlying diseases. Healthcare workers (HCW) should use appropriate means to prevent influenza transmission in healthcare settings, with vaccination considered as the most important recommended measure. At our institution, a significant proportion of HCWs refuse vaccination. As an alternative solution to protect patients from SI, HCWs at our hospital are now obliged to be vaccinated or wear masks during SI epidemics. We propose a method for the quantification of adherence to this recommendation.

## Methods

HCWs vaccinated against SI wear an orange badge with the text "I am vaccinated to protect you". HCWs who are not vaccinated wear a brown badge with the text "I wear a mask to protect you" and must wear a mask during the influenza epidemic (about 3 months) in ward corridors and patient rooms. During the SI epidemic, one investigator audited the observance of recommendations over three periods of two weeks each between January and March 2013 by recording HCWs with an orange badge and HCWs with correct mask wear. To estimate adherence, we calculated: (number of HCWs wearing an orange badge + number of HCWs wearing a mask correctly)/number of HCWs observed = number of compliant HCWs/number of HCWs observed, expressed as a percentage. SI surveillance included active screening of all suspected cases using nasopharyngeal samples analyzed by real-time rtPCR.

## Results

A total of 2937 HCWs were observed: 1171 HCWs with an orange badge and 899 HCWs with a mask, corresponding to an estimated compliance of 70.5% (2070 HCWs) with institutional recommendations. This method can be used for a department or ward and it can be linked to other results, such as the number of nosocomial cases.

## Conclusion

This proposed new method to assess process control of nosocomial influenza transmission measures at the hospital level is simple and easy to use.

## Disclosure of interest

None declared

